# Notes and News

**Published:** 1901-12-07

**Authors:** 


### Notes and News.

At the quarterly court of the governors of the Brompton Hospital, held last week, it was announced that the working drawings of the proposed "country branch," at which the open-air treatment is to be carried on, are in the hands of the committee, but that funds are greatly needed.

Recent advocacy of what we have termed the policy of the tea-kettle as a means of preventing typhoid fever has raised again the old bogie of the unhealthiness of boiled water. Curiously enough certain officers of the R.A.M.C. are stated to be adherents of this faith. Now we know why tea causes dyspepsia. It is all the boiling of the water!

Some interesting results in regard to the protective power of vaccination have already been obtained from the figures collected during the present epidemic. First of all it is stated that with one exception all the cases of small-pox which have occurred in children under ten years of age are unvaccinated, and that out of the 42 such cases there are 29 deaths, all the deaths being unvaccinated. Next it is shown that out of a total of 81 children under 15 years who have been attacked, 57 are unvaccinated and 38 have died. Only one death out of the 38 took place in a vaccinated child, 23 of the 24 vaccinated children having recovered.

A movement is on foot among the metropolitan boroughs to induce the President of the Local Government Board to introduce into Parliament, with all expedition, a Bill authorising sanitary authorities to exercise compulsory control over the movements of the person and clothing of vagrants that have become infected by contact with small-pox, either by means of quarantine in the house, shelter, ward, or other place where they may be found, or by temporary seclusion in quarantine stations to be acquired and maintained by sanitary authorities. There is no doubt there is a great want of some such powers. Much that has been done in certain towns in regard to the isolation of "contacts" has been done quite without regard to law, and both might and would be resisted if it were attempted on a larger scale.

The death is announced of Surgeon-General Harvey, honorary surgeon to his Excellency the Viceroy of India, and Director-General of the Indian Medical Service, C.B., D.S.O., F.R.C.P., LL.D. He was born in 1842, and entered the Bengal Medical Service in 1865. He had seen much service, which, however, had not prevented him from contributing many papers to the medical journals.

The Public Health Committee of the Royal borough of Kensington have recommended the adoption of a system of voluntary notifications by medical men of cases of consumption attended with tuberculous expectoration; payment to medical man of the customary fee for notification; and an arrangement for the bacteriological examination of expectoration suspected to be tuberculous.

The strange correspondence which has been dragging on in the columns of the *Times* for so long in regard to the so-called lachnanthes treatment of consumption has received an interesting addition in the form of a letter from Sir William Broadbent, in which, in addition to exposing some of the absurdities in the statements which have been made on the subject, he shows that the case quoted by Colonel Trench in support of the efficacy of this method was really treated "on open-air lines, resting all day in an open garden, and her dietary consisted of raw meat, underdone steak, chops, etc., milk, eggs," etc. In addition to all this she had certain drugs, to which, of course, the credit is put down. We have all heard of the nourishing nature of "stone broth," a compound made by boiling a pebble. This seems at first sight poor stuff, but to give it a flavour some good shin of beef, vegetables, and various condiments were added, and we believe the result was satisfactory. Nowadays pebbles are spelled l-a-c-h-n-a-n-t-h-e-s.

It is with the most profound regret that we record the death of Sir William MacCormac, Bart, K.C.V.O., F.R.C.S., Surgeon-in-Ordinary to the King. Sir William MacCormac was the eldest son of Dr. Henry MacCormac, of Belfast, where he was born in Belfast in 1836. He was educated in Belfast, Dublin, and Paris. In 1870 his name came prominently before the public when he became surgeon-in chief to the Anglo-American Ambulance in the Franco-German war. He was present at the battle of Sedan, and he related many of his war experiences in his well-known work, "Notes and Recollections of an Ambulance Surgeon." He was surgeon, and of late years consulting surgeon to St. Thomas's Hospital, and also to the French Hospital, and to the Italian Hospital in London. He was President of the Royal College of Surgeons of England for five terms of office, and he was literally covered with foreign honours and decorations. His spirited action in volunteering for South Africa while he was President of the Royal College of Surgeons, and while thus standing at the head of his profession, was highly appreciated in all quarters, and did much, at a time of great anxiety, to restore confidence and to bring comfort to many a home. His death will be felt as a great and almost personal loss by many ranks of society.

